# If you build it, will they come? A comparative landscape analysis of ocelot roadkill locations and crossing structures

**DOI:** 10.1371/journal.pone.0267630

**Published:** 2022-05-03

**Authors:** AnnMarie Blackburn, Amanda M. Veals, Michael E. Tewes, David B. Wester, John H. Young, Randy W. DeYoung, Humberto L. Perotto-Baldivieso

**Affiliations:** 1 Caesar Kleberg Wildlife Research Institute, Texas A&M University-Kingsville, Kingsville, Texas, United States of America; 2 Environmental Affairs Division, Texas Department of Transportation, Austin, Texas, United States of America; Texas State University, UNITED STATES

## Abstract

Wildlife-vehicle collisions can have a substantial influence on the mortality rates of many wildlife populations. Crossing structures are designed to mitigate the impact of road mortality by allowing safe passage of wildlife above or below roads, and connect to suitable areas on both sides of the road. Ocelots (*Leopardus pardalis*) are a federally endangered felid in the United States, with remnant populations of <80 individuals remaining in the Lower Rio Grande Valley of South Texas. Vehicle collisions are the greatest known source of mortality for ocelots in Texas. Crossing structures designed for ocelot use have been implemented throughout South Texas since the 1990s, however, ocelots rarely use them. We compared landscape characteristics between ocelot crossing structures and ocelot-vehicle collision sites. We quantified the spatial distribution of woody and herbaceous cover types surrounding ocelot crossing structures (*n* = 56) and ocelot-vehicle collision sites (*n* = 26) at multiple spatial extents and compared landscape metrics between these location types. The landscape surrounding ocelot crossing structures had 17–22% more open herbaceous cover >1,050 m from the road, and 1.2–5.8 ha larger herbaceous patches >450 m from the road compared to ocelot-vehicle collision sites. Additionally, many crossing structures installed during the 1990’s are situated >100 km away from an extant ocelot population. Results from this study can guide conservation planners to place future road crossing structures in areas more likely to be used by ocelots. Our results also emphasize that reliable scientific data must be used for effective mitigation efforts. In the absence of data, post-installation assessments can improve the placement of future structures.

## Introduction

Transportation infrastructure has grown exponentially in the last several decades, extending the human footprint unavoidably deeper into wildlife habitat. Wildlife-vehicle collisions are now a major source of mortality for wildlife and may have significant impacts on imperiled populations [1]. Road networks also affect wildlife populations by causing habitat loss, fragmentation, and barriers to movement [2, 3]. As traffic volume and speed increase, successful wildlife crossings decrease significantly [4]. Roads that fragment otherwise continuous habitat may lead to small, isolated populations with increased potential for genetic drift and local extinction [4]. Therefore, roads are an obvious threat to the long-term survival of wildlife populations, particularly species with low densities and low fecundity [2].

Reduction of vehicle collisions can prevent significant population decline and improve landscape connectivity, particularly for imperiled species. Ability to disperse and colonize new areas is important for populations to remain viable within fragmented landscapes [4]. Although many wildlife populations do not experience roadkill rates sufficient to affect population size at the geographic level [5], vehicle collisions often represent a significant source of mortality for wild felids. Many populations of felids exist at low densities over a large geographic area and are thus uniquely vulnerable to road mortality. For instance, vehicle collisions are the highest source of mortality for endangered felids, such as the Iberian lynx (*Lynx pardinus*; [6]) and Florida panther (*Puma concolor coryi*; [7, 8]). Road crossing structures can reduce the effects of habitat fragmentation and prevent wildlife-vehicle collisions, particularly for sensitive species with high mortality rates related to road networks [7, 9].

Mitigation using crossing structures typically focusses on two main goals: to connect habitat and wildlife populations fragmented by roads, and to reduce road-related wildlife mortality [2]. Poorly designed or sited measures fail to effectively mitigate the negative effects of roads and can waste funds used for implementation [4]. Ideal wildlife crossing structures should link species to habitat that will enable individuals to meet biological requirements including dispersal, colonizing available areas, and finding mates and other resources [2, 4]. Studies suggest wildlife-vehicle collisions are often spatially aggregated [10] and most frequently occur along roadways near natural vegetation cover [11, 12]. Vehicle collisions of felid species typically occur near preferred habitats and use areas [6, 13–15]. Therefore, placement of wildlife crossing structures to facilitate connection of habitat patches and the broader landscape are key factors to the success of crossing structures [2, 4].

Ocelots (*Leopardus pardalis*) are a medium-sized felid ranging from central South America, through Mexico, with remnant populations in the southern United States (US). Ocelots are federally endangered within the US and state endangered within Texas [16, 17]. Fewer than 80 ocelots remain in the US, restricted to two isolated breeding populations in the Coastal Sand Plain and Lower Rio Grande Valley eco-regions of South Texas [18, 19]. Ocelots are habitat specialists that select for areas with ≥75% thornshrub canopy cover and have movement patterns strongly linked to dense thornshrub [20, 21]. However, land modification from urbanization and agriculture has continued to reduce quality ocelot habitat within the southernmost 13 Texas counties to <1% of the area [22]. With the expansion of human population centers in the Lower Rio Grande Valley, ocelots face greater pressure to survive in an increasingly fragmented landscape [23]. Their fate is exacerbated by the development and expansion of road networks, as previous research suggests that vehicle collisions are one of the highest sources of mortality for ocelots in South Texas, representing about 40% of all mortalities [24, 25].

Population viability models predict that reducing road mortality is the most effective strategy to reduce the likelihood of local extinction for South Texas ocelots [26]. The establishment of crossing structures can aid in reducing road mortality for ocelots as well as promote successful dispersal by providing safer linkages between fragmented habitat patches. In fact, crossing structures were first built specifically for use by ocelots in the South Texas region during the 1990’s [19, 27, J. Young, Jr., *personal communication*]. However, there have been only two documented cases of crossing structure use by an ocelot [28, J. Young, Jr., *personal communication*]. Many currently built crossing structures are >150 km from known ocelot populations, and thus have little effect on road mortality. The lack of effectiveness has raised questions about the criteria for prioritization, siting, and placement of crossing structures for ocelots [19].

Recent studies found that ocelot road mortalities in South Texas are associated with areas of intact thornshrub composed of 30–38% woody cover and woody patches of 3.5 ha [29, 30]. While this information gives conservation planners an understanding of where collisions may occur, it can also be used to evaluate the efficacy of current crossing structures built specifically for ocelots. This study aimed to expand upon the findings of Blackburn et al. [29] by further analyzing ocelot-vehicle collisions in the context of mitigation. Our goal was to understand whether ocelot crossing structures (hereafter crossing structures) occur in areas suitable for ocelots by comparing landscape features at these locations to where ocelots have been killed by vehicles. Specific objectives were to (1) quantify the spatial distribution of land cover types at current and planned crossing structures in South Texas, (2) compare spatial patterns of landscape features at crossing structure locations to ocelot-vehicle collision sites, and (3) assess the average distance between the nearest ocelot crossing structure and ocelot-vehicle collisions sites.

## Materials and methods

### Study area

**Fig 1:**
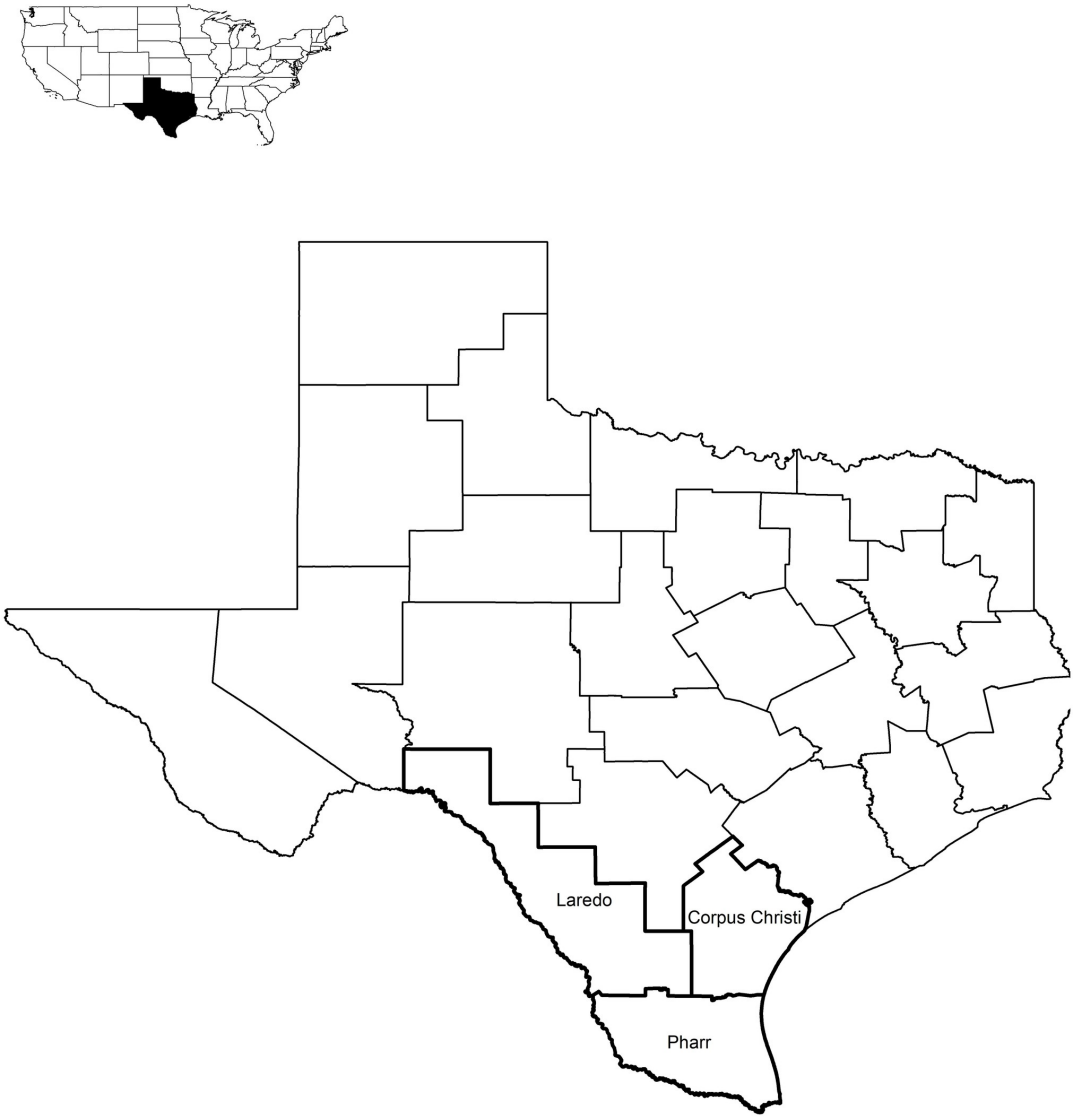
Districts defined by the Texas Department of Transportation, highlighting Laredo, Corpus Christi, and Pharr districts. Boundary data from TxDOT Open Data Portal, original copyright 2018.

The South Texas region includes three of 25 districts defined by the Texas Department of Transportation: the Laredo, Corpus Christi, and Pharr Districts (Fig 1). South Texas is dominated by semi-arid and subtropical climates, with high summer and annual temperatures of 21–23°C and mild winters [31]. This region consists of four major biotic provinces: Chihuahuan (warm desert), Kansan (temperate grasslands), Tamaulipan (subtropical), and Austroriparian (south temperate forests) [31]. Mean annual rainfall for South Texas combined is 660 mm [31]. The Laredo District includes the Chihuahuan, Kansan, and Tamaulipan Biotic Provinces and is dominated by thornshrub forest, grasslands, and pastures [31, 32]. Mean annual rainfall in the western region of South Texas is 592 mm [31]. The Corpus Christi District includes the Austroriparian and Tamaulipan Biotic Provinces, which are dominated by pastures and cultivated crops [31, 32]. Mean annual rainfall in the eastern region of South Texas is 761 mm [30]. The Pharr District lies entirely within the Tamaulipan Biotic Province, which is dominated by grasslands, coastal prairie, live oak (*Quercus virginiana*), thornshrub forest, and cultivated crops [31, 32]. Mean annual rainfall in the southern portion of South Texas is 625 mm [31].

### Data collection

**Fig 2:**
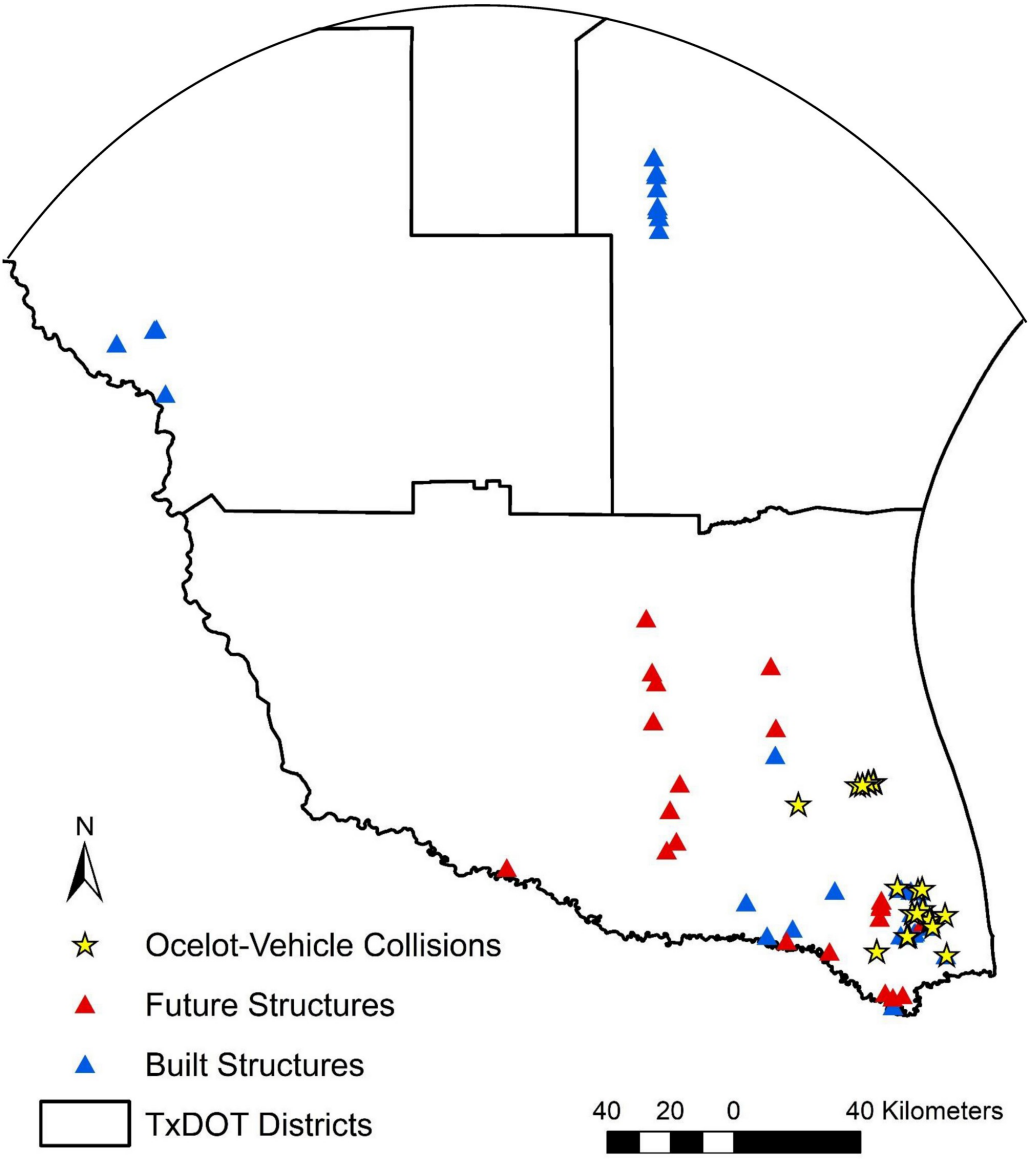
Locations of 26 ocelot-vehicle collisions (stars) and ocelot road crossing structures (triangles) in the Pharr district. Boundary data from TxDOT Open Data Portal, original copyright 2018.

In the 1990’s, the United States Fish and Wildlife Service (USFWS) considered the distribution of ocelots or potential habitat to incorporate the southernmost 30 counties of Texas, approximately [33]. Currently, USFWS considers the ocelot to occur, or potentially occur, in 11 counties [34]. Because of this, federally funded transportation projects were required to assess the impacts of the project activities on endangered species, including the ocelot [J. Young, Jr., *personal communication*]. Several projects within Corpus Christi, Laredo, and Pharr districts were deemed to have a potential to impact ocelots, thus requiring consultation with USFWS [J. Young, Jr., *personal communication*]. Upon consultation, mitigation efforts were required, resulting in road crossing structures. To date, 33 crossing structures have been built primarily for ocelot use [19, 27, J. Young, Jr., *personal communication*]: four in the Laredo District, eight in the Corpus Christi District, and 21 in the Pharr District. Some of these crossing structures have been monitored in the past, though not with the same amount of effort [14, 35]. Resident ocelots are only known to occur within the Pharr District [18, 22], where nine additional crossing structures are committed to be built and 14 more are in the planning and design stages. The current, committed, and planned crossing structures (*n* = 56) are underpasses that consist of round metal culverts, modified concrete box culverts, or undercross bridges (Fig 2; S1 Table).

There were 54 recorded ocelot-vehicle collisions between 1982 and 2017 in the Lower Rio Grande Valley, 32 of which had recorded geographic locations. We removed six ocelot-vehicle collisions due to location inaccuracies or abnormal locations. The final data set included 26 known ocelot-vehicle collision sites: 18 males, seven females, and one unknown sex. This database was compiled largely from incidental roadkill observations reported from the general public and game wardens, as well as ocelots actively being, or previously, monitored.

### Landscape analysis

We used 30-m satellite imagery to evaluate the spatial structure of land cover classes [29]. Specifically, we acquired seven LANDSAT images through the United States Geological Survey Global Visualization Viewer (https://glovis.usgs.gov/) which were taken during the year crossing structure construction was completed, or within two years of estimated completion. Images consisted of four scenes from LANDSAT 5 Thematic Mapper (1994–2009) and three scenes from LANDSAT 8 Operational Land Imager (2017–2018). For evaluation of ocelot-vehicle collision sites, we acquired 15 additional LANDSAT images taken within two years of each vehicle collision, which consisted of 10 scenes (1986–2010) from LANDSAT 5 Thematic Mapper and five scenes (2014–2017) from LANDSAT 8 Operational Land Imager.

We created land-use cover maps by grouping each image into four land cover classes: woody cover, herbaceous cover (i.e., non-woody vegetation), bare ground, and water using unsupervised classification methods [23, 36] in ERDAS IMAGINE 2018 (Hexagon Geospatial, Norcross, GA). We manually digitized crop fields for each image and fused these layers to the classified imagery. Thus, the final imagery included five land cover classes: woody, herbaceous, bare ground, water, and cropland. To assess the accuracy of classification, we generated 200 random points across each image in ArcMap 10.6.1 (ESRI, Redlands, CA), assigned each random point to one of the five classes, and compared the observed cover class to the expected cover class from the classified image using a confusion matrix [37]. We continued accuracy assessments until each image surpassed 85% accuracy [38, 39].

We quantified and evaluated the variation in landscape characteristics within multiple spatial extents surrounding crossing structures and ocelot-vehicle collision sites. Landscape structure can influence animal response (e.g., movement, abundance) at multiple spatial scales, where the scale at which the effect is the strongest is known as the “scale of effect” [40, 41]. To estimate the potential scale of effect, we measured landscape variables within 11 nested spatial extents surrounding crossing structure and vehicle collision sites. Spatial extents increased in radius from 150–1,650 m by 150 m increments. Increments of 150 m were used to create fine-scale spatial increases while including enough pixels in each additional spatial extent to accurately quantify changes in land cover patterns. The largest spatial extent encompassed the maximum average daily movement length (m) by ocelots in South Texas (M. Tewes, Texas A&M University-Kingsville, *unpub*. *data*).

For each crossing structure and ocelot-vehicle collision location, we quantified the amount and distribution of woody and herbaceous cover types by clipping the corresponding classified imagery to each spatial extent. We did not quantify the landscape structure of bare ground cover because of differences in classification strategy between imagery for crossing structure locations and ocelot-vehicle collisions. We calculated seven class-level landscape metrics (Table 1): percent land cover (PLAND, %), patch density (PD, patches/ha), largest patch index (LPI, %), edge density (ED, m/ha), mean patch area (MPA, ha), mean nearest neighbor (ENN, m), and aggregation index (AI, %). These metrics described the amount and spatial distribution of land cover features within each extent and have been used to analyze habitat features of endangered felids [42–44]. Landscape metrics were calculated using FRAGSTATS 4.2 [45].

**Table 1:** Definitions of class-level landscape metrics used in analysis of landscape spatial structure of 26 ocelot-vehicle collision sites and 56 crossing structures in South Texas.

Landscape Metric	Definition
PLAND	Percent land cover (%)
PD	Patch density (patches/ha)
LPI	Largest patch index (%)
ED	Edge density (m/ha)
MPA	Mean patch area (ha)
ENN	Euclidean nearest neighbor (m)
AI	Aggregation index (%)

### Statistical analysis

We used two statistical approaches to compare the seven landscape metrics between crossing structure locations and ocelot-vehicle collision sites [29]. First, we used a sign test [46] to evaluate how frequently one location type has a larger value for a given metric; however, this test does not take into consideration the magnitude of the differences between medians. Second, we used the Kolmogorov-Smirnov test [47] to compare medians of each landscape metric at all spatial extents at crossing structures to ocelot-vehicle collision sites. We report effect sizes as the maximum difference between the empirical distribution functions (D_max_), which ranges from zero to one. Statistical analyses were conducted in SAS version 9.4 (SAS Institute, Cary, NC, USA). Because we conducted 154 Kolmogorov-Smirnov tests for comparisons between vehicle collision and random sites, we also summarized results using Bonferroni’s adjustment to *p*-values [48] for each set of comparisons.

### Distance analysis

Lastly, we aimed to analyze the distance between locations of crossing structures and ocelot-vehicle collision sites. Road segments with high rates of wildlife-vehicle collisions can be an indicator of where mitigation is needed, though it is not always the sole indicator [49]. The crossing structures built in the Laredo and Corpus Christi Districts are several counties away from any known ocelot-vehicle collision site, known resident populations, or even the closest known populations in Mexico. Therefore, these structures have a very low probability of use by ocelots. Our goal was to understand where crossing structures exist spatially in relation to ocelot-vehicle collision sites. For instance, if road mortalities occurred near crossing structures, then the design or location of the structure may need alteration. Conversely, if road mortalities occurred far from crossing structures, then the expenditure of resources might be better directed for mitigation purposes. We evaluated distance separately for three temporal categories of crossing structures: “old” structures built between the 1990s and early 2000s, “recent” structures built between 2015 and 2020, and “future” structures that will be implemented after 2020. We measured the distance between each crossing structure location to the nearest ocelot-vehicle collision site using the ‘near’ tool in ArcMap 10.6.1 (ESRI, Redlands, CA, USA). We present these data as a frequency distribution.

## Results

### Landscape analysis

**Fig 3:**
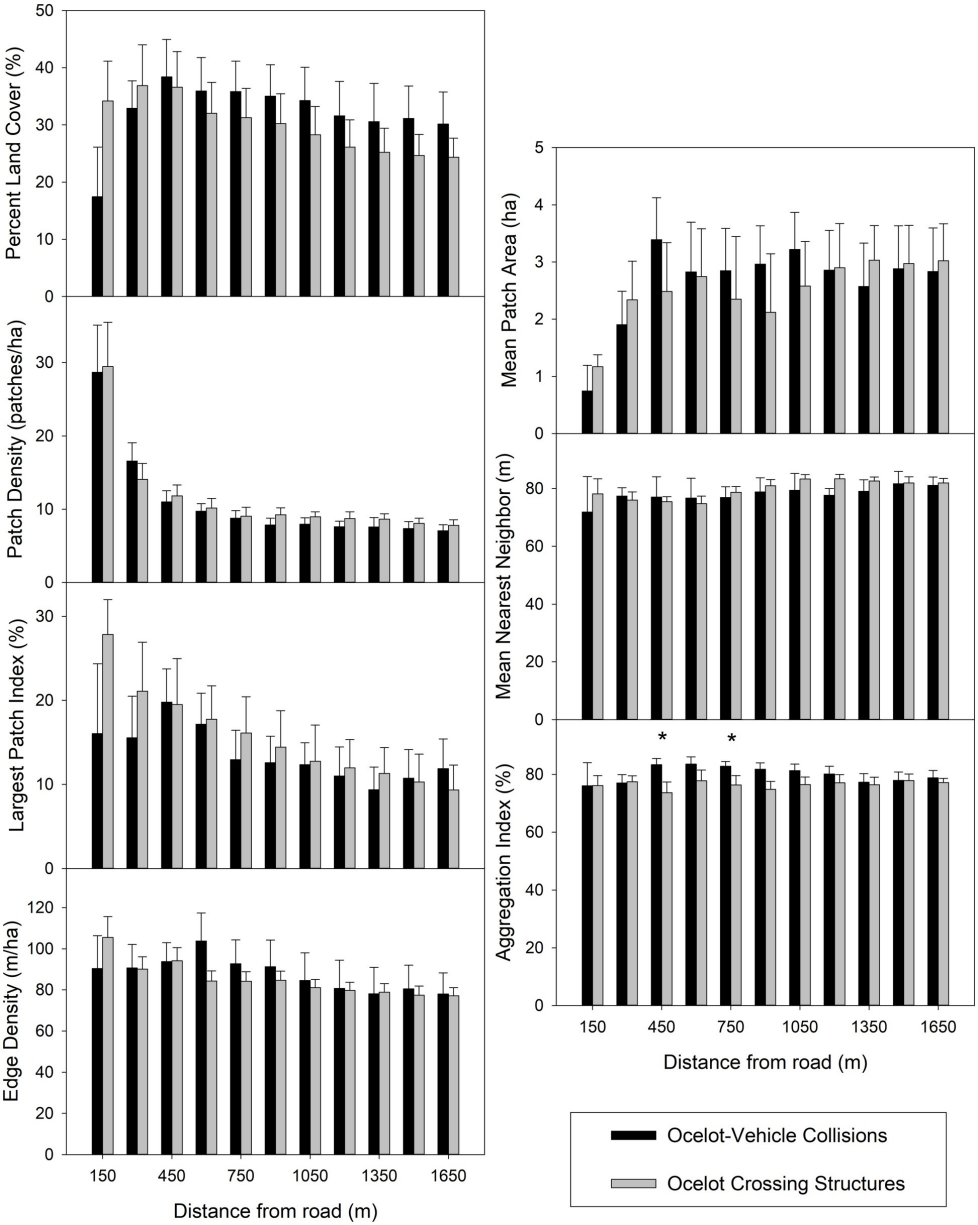
Median values of woody vegetation structure between 26 ocelot-vehicle collision sites and 56 ocelot crossing structures. Asterisks on top of bars indicate significant differences based on Kolmogorov-Smirnov tests.

Woody cover values of PLAND and AI were more likely to be greater at ocelot-vehicle collision sites (sign test: *p < 0*.*06*; Table 2), while values of PD were more likely to be greater at crossing structure sites (sign test: *p < 0*.*01*). Values of LPI, ED, MPA, and ENN were not likely to be greater at either location type (sign test: *p > 0*.*23*). Median PLAND was 16% greater for crossing structure sites at the 150 m spatial extent (D_max_ = 0.30, *p < 0*.*07*; Fig 3), and 4–7% greater for ocelot-vehicle collision sites at spatial extents >450 m (D_max_ < 0.21, *p > 0*.*43*). Nearest neighbor median values were about 5 m shorter at crossing structures at the 1,200 m extent (D_max_ = 0.29, *p < 0*.*08*). Median AI values were 5–10% greater at ocelot-vehicle collision sites at the 450–750 m spatial extents (D_max_ > 0.30, *p < 0*.*06*). There were no statistical differences in median values of PD, LPI, ED, and MPA (D_max_ < 0.26, *p > 0*.*18*). The above effects were not significant following Bonferroni adjustment.

**Table 2:** Results of the sign tests from woody and herbaceous comparisons of ocelot-vehicle collision sites and crossing structures. P-values are relative to ocelot-vehicle collision sites.

	Woody^1^ Sign test	Herbaceous^2^ Sign test
Metric	*p <*	*p <*
PLAND	0.07	1.00
PD	1.00	0.01
LPI	1.00	1.00
ED	0.23	0.07
MPA	1.00	1.00
ENN	1.00	1.00
AI	0.07	1.00

^1^Results from the Kolmogorov-Smirnov tests are in Fig 3

^2^Results from the Kolmogorov-Smirnov tests are in Fig 4

**Fig 4:**
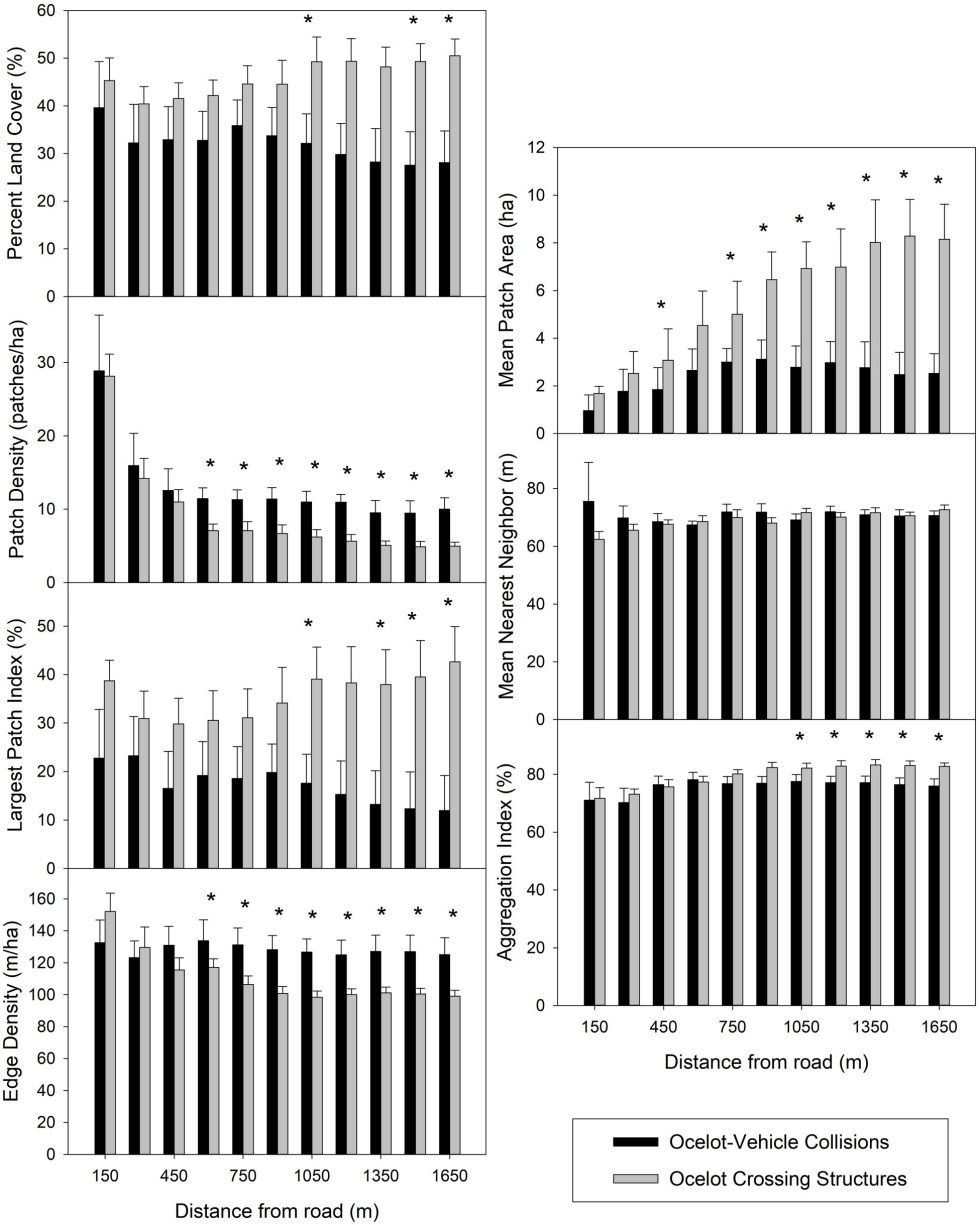
Median values of herbaceous vegetation structure between 26 ocelot-vehicle collision sites and 56 ocelot crossing structures. Asterisks on top of bars indicate significant differences based on Kolmogorov-Smirnov tests.

Values of PD and ED for herbaceous cover were more likely to be greater at ocelot-vehicle collision sites than at crossing structures (sign test: *p < 0*.*06*; Table 2), while values of PLAND, LPI, MPA, and AI were more likely to be smaller (sign test: *p < 0*.*06*). Values of ENN were similar at either location type (sign test: *p = 1*.*00*). Median values of PLAND were 17–22% larger at crossing structure locations than ocelot-vehicle collision locations at spatial extents >1,050 m (D_max_ > 030, *p < 0*.*06*; Fig 4). Median PD values were 4.2–5.0 patches/ha larger for ocelot-vehicle collision sites than ocelot crossing structure location at spatial extents >600 m (D_max_ > 0.35, *p < 0*.*02*). Median LPI values were 21–30% larger at crossing structure locations at the 1,050 m and 1,350–1650 m spatial extents (D_max_ > 0.30, *p < 0*.*05*), and 13% and 23% larger at the 450 m and 1,200 m extents, respectively (D_max_ > 0.29, *p < 0*.*08*). Median values of ED were 16–28 m/ha greater at ocelot-vehicle collision sites (D_max_ > 0.33, *p < 0*.*03*). Median MPA was 1.2–5.8 ha greater at crossing structure locations at 450 m and 750–1,650 m extents (D_max_ > 0.31, *p < 0*.*05*), and 1.9 ha larger at the 600 m extent (D_max_ = 0.29, *p < 0*.*07*). Median AI values were 4.6–6.7% greater at crossing structure locations at spatial extents >1,050 m (D_max_ > 0.31, *p < 0*.*05*). There were no significant differences in median values of ENN (D_max_ < 0.29, *p > 0*.*20*). Using Bonferroni-adjusted p-values, MPA was greater at crossing structure locations at the 1,350 and 1,650 m spatial extents, and PD was greater at ocelot-vehicle collision sites at the 1,350–1,650 m extents.

### Distance analysis

**Fig 5:**
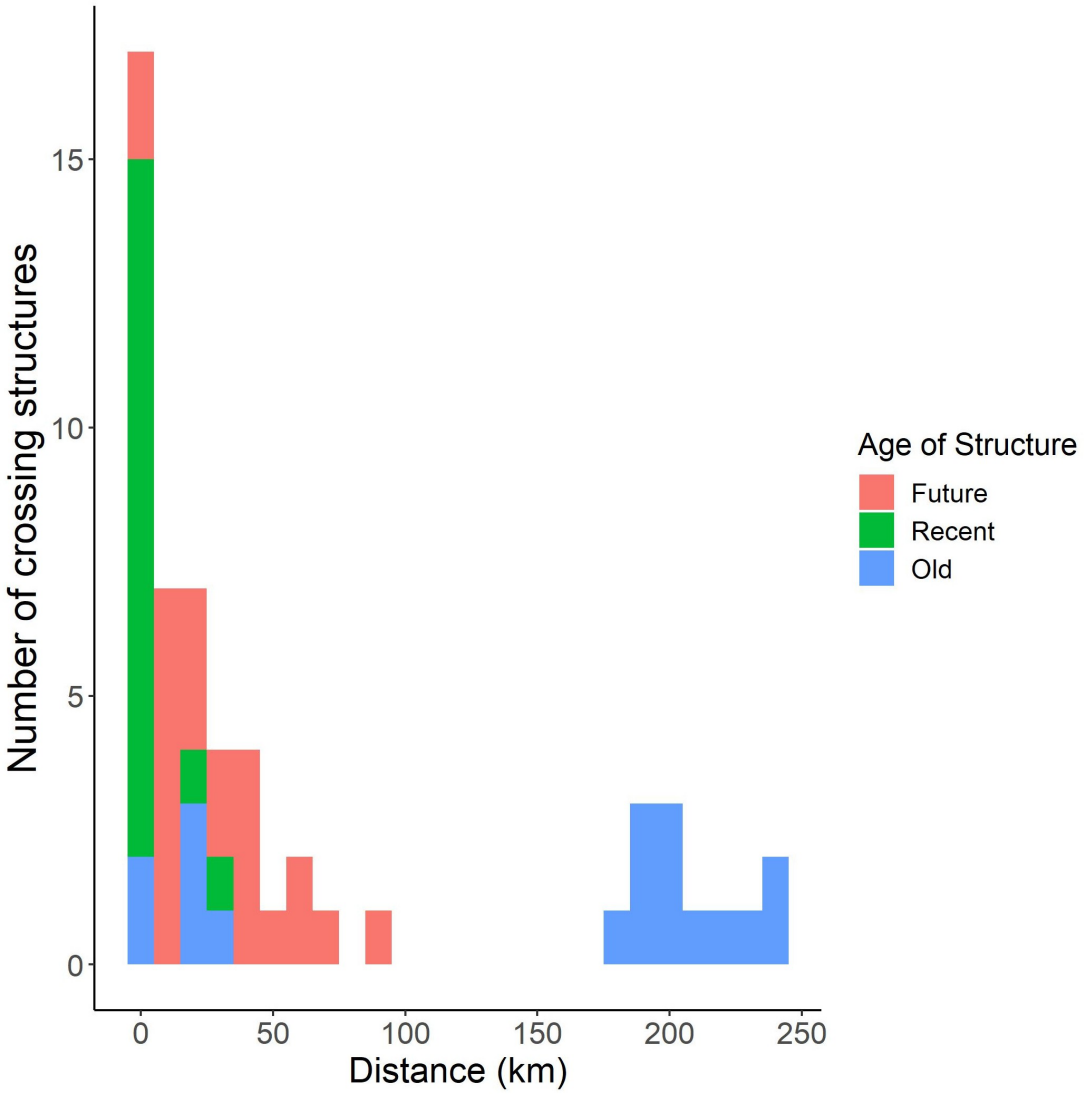
Histogram depicting the distance between each of the 56 crossing structures to the nearest ocelot-vehicle collision site.

The distance between all 56 crossing structures and 26 ocelot-vehicle collision sites ranged from 0.04 km to 240 km (Fig 5). For the crossing structures currently built, the old structures (*n* = 18) had an average distance of 204 km (range = 0.13 km–240 km) and the recent (*n* = 15) structures had an average distance of 4 km (range = 0.04 km–31 km) from vehicle collision sites. The average distance between the 23 future crossing structures and ocelot-vehicle collisions was 28 km (range = 0.11 km–85 km). Seven of the recent crossing structures have been built within 1 km of a vehicle collision, whereas only two of the old crossing structures occur within 1 km. Four future crossing structures will be built >50 km from an ocelot-vehicle collision while the 12 in the Laredo and Corpus Christi Districts >180 km from an ocelot-vehicle collision site.

## Discussion

Wildlife crossing structures can be an effective tool for mitigation against negative effects of road networks on wildlife populations. However, if not properly designed and placed, the rate of successful use may be limited [2]. Ocelots are habitat specialists that prefer interior thornshrub communities in South Texas, and typically select for areas with ≥75% thornshrub canopy cover [20, 21]. Accordingly, the quantity and distribution of land cover influence where ocelots attempt to cross roads [29]. Our results suggest ocelot-vehicle collision sites and crossing structures had similar amounts of woody cover in their surroundings, but collision sites had woody patches that were spaced closer together, similar to habitat patterns ocelots typically select [20]. Ocelot-vehicle collision sites had numerous small patches of herbaceous cover that were irregularly shaped spread throughout spatial extents, while crossing structure locations had fewer but large herbaceous patches (i.e., 3–8.3 ha), and were more uniform in shape. Overall, many crossing structures included in this study are, or will be, in areas that contain a significant amount of uniform herbaceous cover, such as coastal prairies, which may encourage ocelots to avoid such structures.

Ocelot crossing structures in South Texas have been built up to 240 km from ocelot-vehicle collisions or known populations, which may contribute to the lack of use. The 26 ocelot-vehicle collisions analyzed in this study occurred over a 35-year time period, whereas several of the crossing structures have been built within the past five years, and thus were not available in the past. However, the crossing structures from the Corpus Christi and Laredo Districts have been built since the 1990s and are >180 km away from the nearest ocelot-vehicle collision site. No resident, breeding population of ocelots has ever been documented in either district, nor have transients or dispersers, however mitigation efforts have been implemented here due to USFWS distribution maps for the ocelot. The construction of mitigation measures at such distances from a known population does little to aid in ocelot conservation. A cluster of eight ocelot-vehicle collisions has occurred >25 km from the nearest crossing structure, which are from the northernmost ocelot subpopulation that exists within private rangelands [19, 44]. This subpopulation is more secluded from urbanization but not completely without the threat of road-related mortality. Private rangelands contain the largest remaining patches of woody cover (e.g., thornshrub and live oak) in the Coastal Sand Plain and Lower Rio Grande Valley [23], which promotes ocelot recovery efforts. However, with vehicle collisions still a threat to ocelot survival, additional crossing structures in this area could aid in their conservation.

Results from this study suggest that planned future crossing structures are more likely to be used based on surrounding habitat features and proximity to previous ocelot roadkills. Previous studies have analyzed ocelot movements in relation to roads [29, Lombardi et al. *in review*], how roads influence ocelot survival rates [25], and ocelot habitat selection in relation to roads [50]. These studies have highlighted how road-related factors can influence ocelot movement throughout an urbanized landscape and where ocelots cross roads, successfully and unsuccessfully. Blackburn et al. [25] suggested that future mitigation efforts should be focused on lower traffic volume roads rather than high traffic volume roads. Lombardi et al. [*in review*] found that ocelots that were observed to be killed by vehicles had crossed paved roads twice as often as ocelots that were not killed by vehicles. Four planned crossing structures in Pharr District will be on lower traffic volume roads, in the proximate area of previous ocelot-vehicle collisions and known ocelot populations, thus having a higher likelihood of being successful ocelot crossing structures. Additionally, if future ocelot conservation efforts are successful in expanding the current range westward and northward, more of the planned future crossing structures have a potential to be successful.

Ocelot use of crossing structures may be influenced by behavior, in addition to the great distances and lack of cover. Of the known ocelot-vehicle collisions in South Texas, 69% were of males, and the collision sites of male ocelots tended to occur in areas with greater bare ground cover and slightly less woody cover than those of females [29]. This may reflect attempted dispersal movements by subadult and young adult males in search of new territories [24, 51, 52]. Dispersing carnivores can be reluctant to use crossing structures, particularly when moving through unfamiliar areas [53, 54]. Avoidance and low use rates of wildlife crossing structures have also been attributed to unnatural characteristics of most underpasses [55]. Further, surrounding vegetation communities, habitat quality, and human disturbance in the vicinity of crossing structures can influence crossing rates [55]. In our study area, patterns at vehicle collision sites indicated that ocelots were likely attempting to cross roads with a high proportion of woody cover compared to random locations [29]. Although most ocelot crossing structures are located in areas with woody cover, if large herbaceous patches dominate the vicinity, the openness will likely cause ocelots to avoid the area. Many crossing structures designed for ocelots are located in largely open herbaceous areas and are far enough away from known populations or roadkill sites to be beyond the capacity for ocelot dispersal. As such, neither the landscape nor siting is suitable enough for ocelot recovery. Because ocelots are habitat specialists that select dense woody cover, restoring thornshrub adjacent to the opening of, as well as farther out from, the crossing structure on both sides of the road could prove beneficial.

Crossing structures in South Texas were built predominantly for ocelot use, although biologists anticipated other wildlife to also benefit from them. The primary goal is to reduce ocelot-vehicle collisions while also facilitating ocelot movement and dispersal across the landscape to expand their distribution and colonize new areas. Identifying the location of wildlife crossing structures based on road mortality data alone is not always an effective way to reduce wildlife-vehicle collisions; managers should consider the habitat requirements and movement ecology of a target species [2]. For carnivores, crossing structures need to be in areas with sufficient ecological significance, such as within highly used areas or connected to vegetated corridors [54]. It is not beneficial to wildlife conservation to implement crossing structures in areas that will lead to population sinks. Our results suggest that ocelots are likely attempting to cross between habitat patches that are bisected by a roadway; previous studies of other endangered felids have reached similar conclusions [6, 13–15].

Multi-scale habitat-based analyses can provide a useful framework to evaluate land cover when selecting sites for crossing structures. Measuring the effects of landscape structure within the appropriate spatial extent is important to correctly identify the effects of landscape attributes on a particular species, as these attributes may influence species behavior at varying spatial scales [40, 41]. Understanding how the landscape structure may influence the location of ocelot-vehicle collisions, and the scale at which these influences are the strongest, can help decision-makers select the optimal location to implement crossing structures. Wildlife crossing structures can be designed at the project-level, to mitigate road mortality and increase population connectivity along a road segment, or at the landscape-level to promote demographic and genetic connectivity across a larger area through corridors [2]. Such structures for ocelots should not lead to ecological “dead ends” [19]. Instead, they should connect ocelots to broader landscapes with extensive habitat to move freely and meet daily needs [2]. This scale-dependent methodology for siting mitigation measures can also be useful for other species threatened by anthropogenic disturbance. The development of future crossing structures needs to incorporate large spatial scale considerations and future land-use changes in the planning process [2]. Most crossing structures analyzed in this study were installed as mitigation measures to minimize impacts of road related projects on ocelots in areas where ocelots did not, and still do not, occur. Results from this study illustrate the need for science-based decision making by conservation planners to properly assess crossing structure sites to benefit wildlife conservation. The transfer of reliable scientific knowledge to agencies responsible for conservation management is necessary for appropriate mitigation measures. Additionally, overestimating the distribution of endangered species may result in conservation action being taken in places where the species does not occur, thus wasting funds. We encourage conservation planners to prioritize properly located crossing structures by (1) considering the land cover in the area, (2) acknowledging multiple spatial scales, (3) understanding the current distribution of the species, and (4) improving upon structure design. The reduction of road mortality and restoring landscape connectivity in optimal locations are essential to the persistence of the ocelot and other felids in the U.S.

## Supporting information

S1 Table Location of wildlife crossing structures in Corpus Christi, Laredo, and Pharr districts of South Texas. (CSV)
